# Sources of non-compliance with clinical practice guidelines in trauma triage: a decision science study

**DOI:** 10.1186/1748-5908-7-103

**Published:** 2012-10-25

**Authors:** Deepika Mohan, Matthew R Rosengart, Coreen Farris, Baruch Fischhoff, Derek C Angus, Amber E Barnato

**Affiliations:** 1 Department of Critical Care Medicine, The CRISMA Center (Clinical Research, Investigation, and Systems Modeling of Acute Illness), University of Pittsburgh, Scaife Hall, 3550 Terrace Street, Pittsburgh, PA, USA; 2 Department of Surgery, University of Pittsburgh, F1266, 200 Lothrop Street, Pittsburgh, PA, USA; 3 RAND Corporation, Suite 600, 4570 Fifth Avenue, Pittsburgh, PA, USA; 4 Department of Social and Decision Sciences, Carnegie Mellon University, Pittsburgh, PA, USA; 5 Department of Medicine, University of Pittsburgh, Suite 200, 200 Meyran Avenue, Pittsburgh, PA, USA

**Keywords:** Physician decision making, Clinical guidelines, Compliance, Trauma, Triage, Heuristics, Perceptual sensitivity, Signal detection theory

## Abstract

**Background:**

United States trauma system guidelines specify when to triage patients to specialty centers. Nonetheless, many eligible patients are not transferred as per guidelines. One possible reason is emergency physician decision-making. The objective of the study was to characterize sensory and decisional determinants of emergency physician trauma triage decision-making.

**Methods:**

We conducted a decision science study using a signal detection theory-informed approach to analyze physician responses to a web-based survey of 30 clinical vignettes of trauma cases. We recruited a national convenience sample of emergency medicine physicians who worked at hospitals without level I/II trauma center certification. Using trauma triage guidelines as our reference standard, we estimated physicians’ perceptual sensitivity (ability to discriminate between patients who did and did not meet guidelines for transfer) and decisional threshold (tolerance for false positive or false negative decisions).

**Results:**

We recruited 280 physicians: 210 logged in to the website (response rate 74%) and 168 (80%) completed the survey. The regression coefficient on American College of Surgeons – Committee on Trauma (ACS-COT) guidelines for transfer (perceptual sensitivity) was 0.77 (p<0.01, 95% CI 0.68 – 0.87) indicating that the probability of transfer weakly increased as the ACS-COT guidelines would recommend transfer. The intercept (decision threshold) was 1.45 (p<0.01, 95% CI 1.27 – 1.63), indicating that participants had a conservative threshold for transfer, erring on the side of not transferring patients. There was significant between-physician variability in perceptual sensitivity and decisional thresholds. No physician demographic characteristics correlated with perceptual sensitivity, but men and physicians working at non-trauma centers without a trauma-center affiliation had higher decisional thresholds.

**Conclusions:**

On a case vignette-based questionnaire, both sensory and decisional elements in emergency physicians’ cognitive processes contributed to the under-triage of trauma patients.

## Background

Trauma affects one out of five Americans, requiring the expenditure of $400 billion in direct medical costs each year. Regionalization—tiered levels of care that distribute the sickest patients to the highest-intensity hospitals (trauma centers)—reduces mortality and morbidity [1-6]. Well-established clinical practice guidelines in trauma specify when to triage patients to specialty trauma centers [7]. Despite concerted efforts to address known barriers to compliance (*e*.*g*., lack of familiarity with the guidelines), between 30% to 70% of all patients who meet criteria for transfer to a trauma center remain at non-trauma centers (under-triage) [8-11]. At the physician level, existing quality improvement efforts have focused on improving knowledge, modifying attitudes, and removing structural and economic barriers to transfer [12,13]. The extent to which cognitive aspects of physician decision-making contribute to under-triage is unknown.

Based on cognitive theory for discrimination tasks, inappropriate disposition decisions (transfer/not transfer) may result from perceptual sensitivity (the ability to discriminate between patients who do and do not meet clinical practice guidelines for transfer) and/or decisional thresholds (the tendency to err on the side of false positive or false negative decisions) [14]. These two dimensions of decision making have different determinants. Perceptual sensitivity reflects both physicians’ knowledge of the clinical practice guidelines as well as intuitive judgments (heuristics) about which patients meet those guidelines. Decisional thresholds reflect variables such as attitudes towards the guidelines, incentives, and organizational norms.

Signal detection theory, a basic decision science method, allows the measurement of these two dimensions of decision making by parsing performance into sensory and decisional components [15]. We used signal detection theory to analyze emergency physician responses to a case vignette-based questionnaire. The objective of this study was to assess whether decision making in trauma triage primarily reflected physicians’ perceptual sensitivity or their decisional thresholds. Correctly attributing the source of non-compliance with clinical practice guidelines would allow us to design more effective quality improvement interventions.

## Methods

Triage of trauma patients can occur either in the field or after an emergency physician has evaluated the patient in the Emergency Department (ED) of a non-trauma center. The American College of Surgeons – Committee on Trauma (ACS-COT) publishes guidelines specifying injuries that warrant transfer to a trauma center. They recommend the transfer of all patients with moderate to severe injuries, defined as either an injury considered to be ‘life-threatening or critical’ or an Injury Severity Score >15. They recommend that emergency physicians make their transfer decision based on information obtained from a history, physical exam, and chest and pelvis x-ray. We conducted a web-based cross-sectional survey among a convenience sample of United States (US) physicians who worked in the EDs of non-trauma centers, and used responses to clinical vignettes, based on case histories, to quantify physicians’ perceptual sensitivity and decisional threshold for trauma triage.

### Development of questionnaire

#### Conceptual model

**Figure 1:**
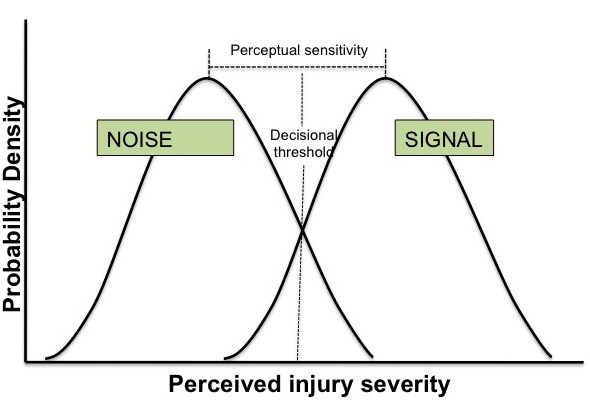
Signal and noise distribution in theory.

Comparing physicians’ decisions to ACS-COT guidelines can lead to four possible outcomes as shown in Table 1: transferring a patient who meets the guidelines for transfer (true positive or ‘hit’); not transferring a patient who meets them (false negative or ‘miss’); transferring a patient who does not meet them (false positive or ‘false alarm’); or not transferring a patient who does not meet them (true negative or ‘correct rejection’) [15]. In contrast to more familiar indices of performance that also rely on 2x2 tables for exposition, such as sensitivity and specificity, signal detection theory parses sensory from decisional factors influencing performance [14]. As depicted in Figure 1, physicians perceive injuries as falling along a continuum ranging from very minor to severe. The ‘signal’ distribution shows their perception of severity for patients who should be transferred according to the guidelines; the ‘noise’ distribution shows their perception of patients who should not be transferred according to the guidelines. The further apart these distributions, the greater their *perceptual sensitivity*. The *decisional threshold* is the point on the continuum above which physicians transfer patients. If perceptual sensitivity remains constant, then as the decisional threshold moves to the right, physicians tend to err toward false negative decisions (not transferring patients with the ‘signal’ of serious injury). As it moves toward the left, they tend to err toward false positive decisions (transferring patients with the ‘noise’ of less serious injuries) [15]. 

**Table 1:** 2x2 table used to categorize identification decisions

	**Meets reference standard**	**Does not meet the reference standard**
**Decision to transfer**	True positive (Hit)	False positive (False alarm)
**Decision not to transfer**	False negative (Miss)	True negative (Correct reject)

### Clinical vignettes

**Figure 2:**
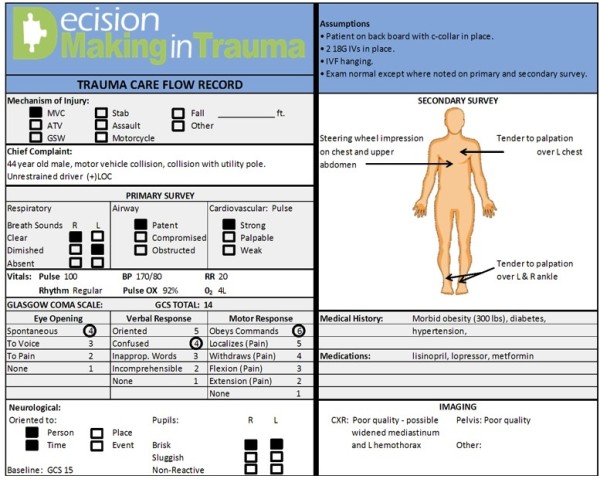
Example of case vignette.

Using the ACS-COT guidelines for the transfer of trauma patients as our reference standard, we constructed 50 clinical vignettes based on the case histories of patients admitted to the University of Pittsburgh Medical Center – Presbyterian Hospital trauma service [1]. Each vignette included all the information the physician would ordinarily obtain from a history, physical exam, chest and pelvis x-ray (*i*.*e*., all the information the ACS-COT considers necessary to triage the patient). We presented the information in the format of a completed trauma care flow sheet, a method recommended by the ACS-COT to standardize the capture of pertinent data. Figure 2 depicts an example of a case vignette. By design, one-half the vignettes met ACS-COT criteria for transfer (mean Injury Severity Score [ISS] 21, range 9–48) and one-half did not (mean ISS 2.5, range 1–4), based on independent review by three trauma surgeons (kappa score for agreement = 0.85). We systematically varied the complexity of the cases to encompass the range of possible triage decisions. Thirty-three vignettes described a blunt injury mechanism and seventeen a penetrating mechanism. We designed the stimuli so that age, gender, and mechanism of injury were unrelated to injury severity or need for transfer. 

### Clinical vignette pre-testing

To ensure that our vignettes and procedures were clear and without leading information, we conducted one round of pretests using cognitive interviews with University of Pittsburgh fellows and faculty who were board-eligible or certified in EM (n = 9). After revising our materials, we conducted additional cognitive interviews with emergency physicians who practiced in non-trauma centers in western Pennsylvania (n = 17). As a result of this second round of testing, we shifted from forced-choice options to free-response text boxes to avoid the priming that fixed options suggested. We also reduced the set of vignettes to 30, after finding that some physicians could not stay fully engaged while evaluating all 50.

### Physician sample

We recruited physicians at a national meeting of the American College of Emergency Physicians (ACEP) in Fall 2010, using a booth in the Exhibition Hall. We designed the study to capture a moderate effect size of 0.15 for the association between clinical practice guidelines and transfer decisions in a regression model with two independent variables (α = 0.05, 1-β=0.80) [16]. Anticipating a 25% response rate, we recruited 280 physicians. Physicians eligible for participation had completed residency and cared for adult patients in the ED of either non-trauma centers or Level III/IV trauma centers in the United States. Those who completed the questionnaire received a $100 payment.

The University of Pittsburgh Institutional Review Board study reviewed and approved the study.

### Study protocol

Participants completed the web-based protocol at their convenience. After logging in, they read a brief passage describing the task, completed a demographic survey, and evaluated the vignettes.

The demographic survey included questions about age, gender, race, educational background (board certification, Advanced Trauma Life Support (ATLS) certification, years since completing residency), and practice environment (hospital trauma designation, affiliation with a Level I/II trauma center, affiliation with an EM residency program, size of the community served).

The evaluation portion had five blocks of six randomly ordered case vignettes, with each block containing two cases with moderate-severe injury severity/blunt mechanism, one with moderate-severe injury/penetrating mechanism, two with minor injury/blunt mechanism, and one with minor injury/penetrating mechanism. Physicians used a free-response text box to answer the question, ‘What would you do to manage the patient?’ The instructions asked them to provide information about treatment, interventions, and disposition. They had two weeks to complete the survey.

### Analysis

#### Descriptive summary of responses

We coded each free-text response to identify management decisions related to: disposition, consults, imaging, medications, laboratory studies, procedures, and resuscitation efforts. To assess inter-rater reliability, two coders evaluated a random sample of 10% of the management decisions. We found very high agreement for all six types of management decisions (kappa 0.81–0.94) [17]. We used logistic regression models, clustered by physicians, to estimate the predicted probability that physicians would make types of management decisions for minor and moderate to severe cases.

We then scored each response dichotomously as compliant or non-compliant with ACS-COT guidelines. For patients with moderate to severe injuries, we defined compliance as: transferring the patient to a trauma center without obtaining additional testing; transferring the patient after performing additional testing contingent on not delaying the transfer; or consulting trauma surgery (assuming that the surgeon would ensure the appropriate triage). For patients with minor injuries, we defined compliance as: not transferring the patient to a trauma center; transferring a patient to a trauma center contingent on identifying further injuries; or consulting trauma surgery.

### Physician performance

We analyzed performance in two ways. First, we calculated adherence to standard ACS-COT triage benchmarks. The ACS-COT defines under-triage as the proportion of patients with moderate-severe injuries who are not transferred. It defines over-triage as the proportion of transferred patients who have minor injuries [7]. We summarized rates of under- and over-triage for each physician.

Second, we used a regression-based approach to signal detection theory to estimate physicians’ perceptual sensitivity and decisional threshold. With adequate trials (≥100) [18], one can estimate an individual’s perceptual sensitivity and decisional threshold directly from their decisions. Where the cognitive burden of a task limits the number of trials, as it did here, one can use a regression-based approach to estimate the parameters and error estimates [19].

(1) $$\text{Perceptual Sensitivity}\;\left(\text{d}\right)=\text{logit}\left(\text{Hit Rate}\right)–\text{logit}\left(\text{False Alarm Rate}\right)=\;\text{ln}\left(\text{Hit Rate}\right)–\;\text{ln}\left(\text{Miss Rate}\right)–\;\text{ln}\left(\text{False Alarm Rate}\right)+\;\text{ln}\left(\text{Reject Rate}\right)$$

(2) $$\begin{array}{l} \text{Decisional Threshold}\;\left(\text{c}\right)=-\text{logit}\left(\text{False Alarm Rate}\right)\\ =-\text{ln}\left(\text{False Alarm Rate}\right)+\text{ln}\left(\text{Reject Rate}\right) \end{array}$$

When these two equations are combined, they form the logistic regression model:

(3) $$\text{logit p}\left(\text{Y}=1|\text{X}\right)=-\text{c}+\text{dX}$$

where Y is the disposition decision and X is the reference standard [20]. The fit of logistic models approximates signal detection models, but with a scale π/√3 times larger than the Gaussian distribution used in signal detection theory [20].

We fit a model for each physician, predicting the log-odds of his or her disposition decisions (dependent variable) using an intercept and a regression weight on the ACS-COT guidelines for transfer (independent variable). The model’s intercept predicts disposition decisions in the absence of any information, hence provides an estimate for the decisional threshold [20]. The regression weight on the ACS-COT guideline variable represents perceptual sensitivity, showing the degree of reliance on guidelines (implicitly or explicitly) when making disposition decisions [20]. We scaled our estimates by 1.8 to approximate the standard deviation units of signal detection values.

A perceptual sensitivity estimate greater than zero indicated a positive association between the guidelines and decisions to transfer patients. A negative decisional threshold suggested a tendency to transfer trauma patients (*i*.*e*., effectively preferring false positives to false negatives), and a positive value suggested a tendency not to transfer. As accuracy approached the limit (15 of 15 true positive or true negative cases transferred appropriately), the estimation procedure failed. We corrected for perfect scores by converting proportions of 0 and 1 to 1/2N and 1-1/2N (where N equaled the total number of trials) respectively [21].

Applying the ACS-COT benchmarks for triage resulted in the following relationships.

Physicians who achieved <5% under-triage had:

(4) $$\text{Perceptual Sensitivity}>\text{logit}\left(0.95\right)+\text{Decisional Threshold}$$

Physicians who achieved <50% over-triage had:

(5) Perceptual Sensitivity>0

In subsequent analyses, we used a random effects logistic regression model to estimate the perceptual sensitivity and decisional threshold for the entire cohort, adding mechanism of injury (blunt versus penetrating) as an additional predictor of physician disposition decisions. We also used Student’s t-test and Spearman correlations as appropriate to estimate the influence of physician characteristics (age, gender, race, board certification, ATLS certification, years since completing residency), and practice environment (hospital trauma designation, affiliation with a Level I/II trauma center, affiliation with an EM residency program, size of the community served) on perceptual sensitivity and decisional thresholds, and tested these characteristics in a multivariable linear regression model.

## Results

### Physician sample

**Figure 3:**
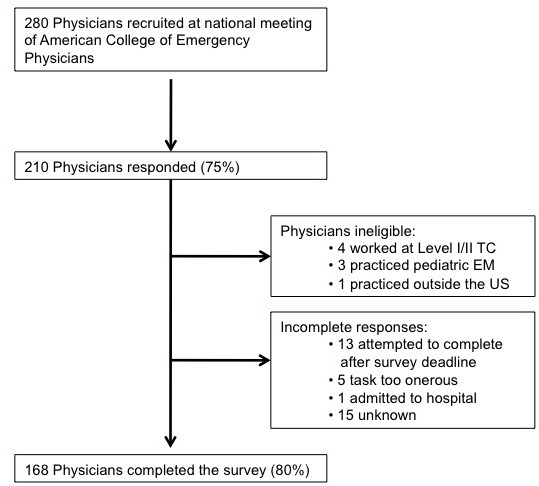
Physician subject enrollment and participation rates.

Participant enrollment and response rates are shown in Figure 3. Among the 280 physicians recruited at ACEP, 210 logged on to the website (75%), of whom 202 met eligibility after reviewing their educational and practice background information. Of the 202 eligible physicians, 168 (80%) completed the questionnaire. The mean age of the physicians who completed the survey was 41.5 years (SD = 9.55). 157 (93%) were board-certified in Emergency Medicine and 125 (74%) certified in Advanced Trauma Life Support. 48 (29%) worked in a hospital affiliated with a level I/II trauma center. Participant characteristics are shown in Table 2.

**Table 2:** Demographic characteristics of physicians

**Variable**	**Value**
Age, mean (SD)	41.5 (9.55)
Gender, n(%)	
Male	141 (84)
Female	27 (16)
Race, n(%)	
Caucasian or white	127 (76)
African American or black	8 (5)
Hispanic or Latino	9 (5)
Asian	20 (12)
Native American	1 (0.6)
Pacific Islander	2 (1.2)
Other/undocumented	1 (0.6)
Primary specialty, n(%)	
Emergency Medicine	157 (93)
Family Practice	7 (4)
Internal Medicine	3 (1)
Other	1 (1)
Years since completing residency, mean (SD)	9 (9.25)
ATLS certified, n(%)	
Yes	125 (74)
No	43 (26)
Years since ATLS certification, mean (SD)	3 (6.6)
Designation of hospital where physician works, n(%)	
III	31 (18)
IV	4 (2)
Non-trauma center (non-TC)	133 (79)
Affiliation of the hospital with a TC (if working at a non-TC), n(%)	
Yes	48 (29)
Affiliation of the hospital with an EM residency program, n(%)	
Yes	26 (15)
Size of community served by hospital, n(%)	
>1 million	19 (11)
250,000 – 1 million	54 (32)
200,000 – 249,999	76 (45)
2,500 – 199,999	18 (11)
<2,500	1 (0.6)

### Analysis

#### Descriptive summary of responses

On average, physicians spent 2.3 minutes (SD = 2.3) per vignette in reading and responding, and listed 2.9 decisions (SD = 1.1; range 1–6). For example, one vignette involved a patient sustaining a fall from a 15-foot balcony, who presented with seizure-like activity. We had categorized the patient as having a moderate to severe injury, based on the ACS-COT guidelines. Participant 110 described this management strategy: order anti-epileptic medications, perform an intubation, obtain a CT of the head and C-spine, and transfer the patient to a trauma center. We coded that response as not compliant with the guidelines because he/she delayed transfer to obtain a CT scan. A summary of types of decisions made for minor and moderate to severe cases is presented in Table 3.

**Table 3:** Predicted probability of management decisions made for patients with minor and moderate to severe injuries

**Decisions**	**Patients with minor injuries**		**Patients with moderate-severe injuries**	
	**Not transferred**	**Transferred**	**Not transferred**	**Transferred**
**Disposition**	37%	-	54%	-
**Consults**	23%	5%	36%	23%
**Imaging**	91%	46%	88%	38%
**Medications**	38%	35%	33%	30%
**Labs**	33%	21%	29%	18%
**Procedures**	32%	41%	57%	66%
**Resuscitation**	7%	15%	48%	28%

### Physician performance

The median under-triage rate among physicians was 80% (Inter-Quartile Range [IQR] 40–97%). In other words, physicians expeditiously transferred 20% of patients meeting the guidelines for transfer. The most common reason for under-triage was delaying transfer to obtain additional imaging. The median over-triage rate was 29% (IQR 0–47%). In other words, 71% of patients transferred met the reference standard. Sixteen participants (9%) did not transfer any patients, but consulted specialists at their own institutions for further management of all cases.

**Figure 4:**
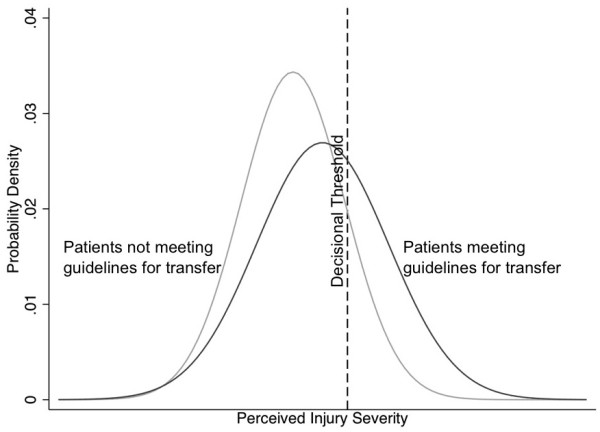
Signal and noise distribution in practice.

In the logistic model for the entire cohort, the regression coefficient on ACS-COT guidelines for transfer (perceptual sensitivity) was 0.77 (p<0.01, 95% CI 0.68–0.87) indicating that, on average, the probability of transfer weakly increased as the ACS-COT guidelines would recommend transfer. The intercept (decision threshold) was 1.45 (p<0.01, 95% CI 1.27–1.63) indicating that, on average, participants had a conservative threshold for transfer, erring on the side of not transferring patients. Estimates of physician perceptual sensitivity and decisional threshold are graphically presented in Figure 4.

**Figure 5:**
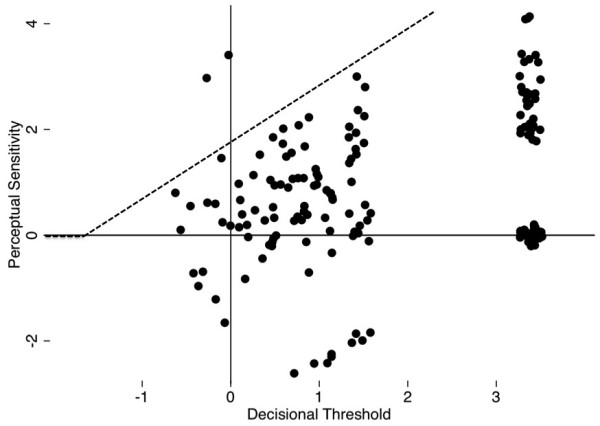
**Individual physicians’decisional thresholds and perceptual sensitivity.** The dotted grey lines indicate the decisional threshold and perceptual sensitivity above which physicians would meet the ACS-COT benchmarks for triage.

As shown in Figure 5, there was significant between-physician variability in perceptual sensitivity and decisional thresholds. 72 (43%) physicians had a perceptual sensitivity estimate greater than 1, indicating better than moderate ability to discriminate between patients who did and did not meet guidelines for transfer. 75 (45%) physicians had a decisional threshold estimate less than 1.45, demonstrating a lower tolerance for errors on the side of not transferring patients. The ACS-COT recommends that physicians at non-trauma centers transfer 95% of patients with moderate-severe injuries, even if that means that up to 50% of all transfers have minor injuries [7]. Given the limited number of cases on our questionnaire, meeting these benchmarks required perfect ability to identify patients who met the guidelines for transfer. Only two physicians (1%) did so. 

Including mechanism of injury in the analysis revealed that cases with penetrating (versus blunt) injuries were more likely to be transferred (OR 1.73, p<0.01, 95% CI 1.46–2.03), even though they were no more (or less) likely to meet ACS-COT guidelines in the sample vignettes.

Physician characteristics (*e*.*g*., gender, training) did not influence perceptual sensitivity. Men had higher decisional thresholds than women (p = 0.01). Physicians with board certification in Emergency Medicine compared to physicians with board certification in Internal Medicine or Family Practice (p = 0.03), and physicians working at non-trauma centers without an established affiliation with a trauma center compared to physicians working at non-trauma centers with an affiliation (p<0.01) had higher decisional thresholds. Two of these associations (gender and affiliation of their hospital with a trauma center) remained significant in the multivariable linear regression model.

## Discussion

In a vignette-based study with 168 emergency physicians, we used signal detection theory to quantify the perceptual sensitivity and decisional threshold of physicians making trauma triage decisions. We found that cognitive processes in physician decision-making may contribute to persistent rates of under-triage in trauma triage. When responding to the cases, many physicians in the study had low perceptual sensitivity, making triage decisions only weakly related (if at all) to the ACS-COT standard. Additionally, most had a high decisional threshold for transfer, systematically erring on the side of not transferring patients to regional trauma centers.

Many epidemiological studies have shown that patients with moderate to severe injuries are routinely under-triaged [8-11]. However, those studies have typically focused on patient-level determinants of variability in triage decisions. Chang *et al*. have described age as a determinant of triage decisions [10]. Macias *et al*. posit that age, co-morbidities, and severity of injury influence the triage of patients with traumatic spinal cord injuries to trauma centers [8]. We used signal detection theory, a basic decision science method, to determine how physician cognitive processes might influence rates of under-triage. Although rarely used for this purpose, signal detection theory has the advantage that it distinguishes between sensory and decisional components of decision making. For example, by using signal detection theory to analyze nursing risk assessments for patients admitted to hospital, Thompson *et al*. have shown that time–pressure and clinical experience influence variability in decision making through different mechanisms. We hypothesized that signal detection theory would allow us to identify reasons for non-compliance with clinical practice guidelines. Better understanding of the cognitive processes responsible for under-triage would provide valuable information for the design of future quality improvement interventions [22].

We presented physicians with a series of case vignettes that required a range of triage decision-making. We found that when answering the case vignettes most physicians demonstrated limited perceptual sensitivity, as shown in Figure 4. The ACS-COT uses ATLS, an educational program that operationalizes the clinical guidelines, as one of its primary tools to standardize the treatment of trauma patients. However, although most physicians in our sample had received ATLS certification, the regression weight on the guidelines in our models suggested that triage decisions on this questionnaire corresponded only weakly with the ACS-COT criteria for the transfer of patients.

Patterns in the responses suggested potential explanations for the limited perceptual sensitivity demonstrated by these physicians. For example, as shown in Table 3, among one-half of the under-triaged patients, physicians correctly identified the patient as meeting the standard for transfer. However, they delayed transport to obtain additional diagnostic imaging. Acquisition of this imaging suggested triage decision-making consistent with the disjunction effect. As described by Shafir and Tversky, people have difficulty making decisions in the context of complex uncertainty. One manifestation of that difficulty is the pursuit of non-instrumental information, which appears relevant but if available would not impact decision making [23,24]. In effect, the information from imaging acquired for patients with moderate to severe injuries would have had negative value, as it reduced the chances of successful treatment.

Similarly, the greater likelihood of transferring patients with penetrating (rather than blunt) injury suggested reliance on the representativeness heuristic. Formally equivalent problems should provoke equivalent judgments. In other words, people should estimate the probability of x belonging to set A in the same way that they estimate the probability of y belonging to set A. However, Kahneman and Tversky have shown that when people rely on the representativeness heuristic to make judgments, they substitute the perceived similarity of x or y to other objects in set A for that probability estimate [25]. In this context, cases with blunt injuries seemed less likely to need tertiary center care as otherwise equivalent cases with penetrating injuries. This hypothesis would also explain patterns of patient-level variability in triage. For example, if physicians judge severe trauma as the purview of young men, they may systematically under-triage elderly patients, as described by Chang *et al*. and Macias *et al*. [8,10].

Figure 4 also demonstrates that the group of physicians who responded to the questionnaire had a high threshold for transfer. To target physicians’ decisional thresholds, the ACS-COT has relied on trauma systems, voluntary networks of local community hospitals that associate with high-volume trauma centers [13]. Through outreach and accreditation programs, it has urged physicians at non-trauma centers to have a low decisional threshold in order to minimize under-triage. In other words, it advocates transferring as many patients as necessary to ensure the capture of all those with moderate to severe injuries, even at the cost of increasing over-triage [7]. Despite these efforts, our sample of physicians preferred to err on the side of minimizing over-triage. Free-text comments by participants suggested that their decisional thresholds reflected conscious negative attitudes towards transferring patients, invoking issues such as resentment towards guidelines in general and distaste at relinquishing control over patient care. The finding of lower decisional thresholds among physicians at hospitals with an established trauma center affiliation suggested that organizational norms or incentives may play roles as well. For example, hospitals without these affiliations may encourage their physicians to avoid transferring patients out of network to prevent the loss of revenue.

Current quality improvement efforts in trauma assume that the same barriers to compliance with clinical practice guidelines affect all physicians equally. However, the individual performance differences revealed in Figure 5 suggest the need for a more nuanced approach. For example, some physicians had a high threshold for transferring patients, and an above average ability to discriminate between patients with minor and moderate-severe injuries. These doctors seemed to at least partially compensate for their apparent unwillingness to transfer patients through skill at distinguishing between those who really did ‘need’ transfer and those who did not. Other physicians had a threshold for transferring patients set around zero and a lower than average perceptual sensitivity. These doctors seemed to at least partially compensate for their apparent inability to distinguish between those who did and did not ‘need’ transfer by being more willing to transfer everyone.

Physicians with decisional thresholds biased away from transfer and with above-average perceptual sensitivity would most likely benefit from an intervention that recalibrated their decisional threshold, perhaps by addressing their concerns about transferring patients or creating financial incentives for the appropriate transfer of patients. In contrast, exposing the second type of physician with a decisional threshold already set around zero to that same intervention might have unwanted consequences. Specifically, increasing their willingness to transfer patients would increase over-triage, and could impose a burden on level I centers. An analysis of triage patterns in Pennsylvania by Mohan *et al*. demonstrates that simply shifting decisional thresholds to achieve ACS-COT targets for triage would result in a five-fold increase in transfers to trauma centers [11]. Moreover, non-trauma centers would lose an important source of revenue and the opportunity to provide care for patients in their community. Instead, these physicians might benefit from a strategy that modified their heuristics, perhaps through training with stimuli like the vignettes used in this study [26].

Our study had several limitations. First, we used vignettes to measure physicians’ perceptual sensitivity and decisional threshold, which did not replicate the time or organizational pressures of clinical decision-making, and with less information than may be available in real-life. Evidence suggesting that case vignettes can predict physician practice patterns comes primarily from the outpatient setting. For example, Peabody *et al*. demonstrated that physicians’ management of ‘paper patients’ with common clinical conditions, like lower back pain, corresponded to their management of real patients [27,28]. Our specific vignettes have the content validity that comes from basing them on the case histories of patients treated at the University of Pittsburgh Medical Center. Additionally, the vignettes were extensively pretested. However, we have no knowledge of whether physician decision-making in response to a case vignette-based questionnaire corresponds to actual practice patterns. An alternative approach, and a possibility for future research, might be using an administrative dataset to calculate physicians’ perceptual sensitivity and decisional threshold based on actual triage decisions.

Second, we used the ACS-COT guidelines as our reference standard for transfer, despite flaws that might affect acceptance by physicians: all the stakeholders did not participate in their design [29]; the definition of over-triage creates an association between prevalence of injury and performance [29], and the benchmarks may lack feasibility [11]. Yet, robust observational data supports their overall validity. MacKenzie *et al*. among others, have shown that patients meeting ACS-COT criteria for transfer have better outcomes when treated at trauma centers [1]. Moreover, their widespread dissemination makes them the *de facto* standard for decision making among physicians, regardless of their potential failings.

Third, we used a non-representative sample of cases on our questionnaire to allow the use of signal detection theory. We did systematically vary the complexity of the cases to elicit the range of decisions that physicians would perform in practice. However, we used a much higher proportion of cases with moderate to severe injuries than physicians would see in practice. We speculate that the absence of non-trauma cases made the triage task easier than the one faced by physicians in their EDs. However, the higher base rate of severe injuries may have altered customary practice patterns. Birnbaum has shown that the predicted effect of the base rate on signal detection estimates depends on the theory of judgment used [30]. We therefore have no clear feeling for how this bias would have affected the patterns observed here. As a test of possible learning effects and base rate influence on the parameter estimates, we compared performance on the first half of the study relative to the second half and found no differences.

Finally, we recruited physicians at a national meeting of emergency medicine physicians, which might limit the generalizability of our observations. However, because physicians attending academic meetings likely have greater knowledge of current clinical practice guidelines, we assume that any bias introduced by our sampling frame would be in the direction of better practice.

## Conclusions

Under-triage persists despite comprehensive efforts by governmental and professional organizations to standardize trauma practice. In responses to a case vignette-based questionnaire, we found that under-triage can result from cognitive processes used in physician decision-making. Further research is required to determine how cognitive aspects of physician decision-making affects the triage of patients in real practice, as well as how best to intervene.

## Competing interests

The authors declare that they have no competing interests.

## Authors’ contributions

DM conceived of the study, participated in the design, acquired the data, analyzed and interpreted the data, and drafted the manuscript. MRR participated in the design of the study, analyzed and interpreted the data, and revised the manuscript. CF conceived of the study, participated in the design, analyzed and interpreted the data, and revised the manuscript. BF conceived of the study, participated in the design, analyzed and interpreted the data, and revised the manuscript. DCA participated in the design of the study, analyzed and interpreted the data, and revised the manuscript. AEB participated in the design of the study, analyzed and interpreted the data, and revised the manuscript. All authors read and approved the final manuscript.
